# ALK1 Signaling in Human Cardiac Progenitor Cells Promotes a Pro-angiogenic Secretome

**DOI:** 10.33696/signaling.5.119

**Published:** 2024

**Authors:** Michayla Moore, Sergey Ryzhov, Douglas B. Sawyer, Carlos Gartner, Calvin P.H. Vary

**Affiliations:** 1 Center for Molecular Medicine, MaineHealth Institute for Research, MaineHealth 81 Research Drive, Scarborough, Maine, USA; 2 Graduate School of Biomedical Science and Engineering, University of Maine Orono, Maine, USA

**Keywords:** Activin a receptor-like kinase 1 (ALK1), Bone morphogenetic protein-9 (BMP9), Human highly proliferative cells (hHiPCs), Endothelial cell tube formation, Sclerostin

## Abstract

Pro-angiogenic paracrine/autocrine signaling impacts myocardial repair in cell-based therapies. Activin A receptor-like type 1 (*ACVRL1*, ALK1) signaling plays a pivotal role in cardiovascular development and maintenance, but its importance in human-derived therapeutic cardiac cells is not well understood. Here, we isolated a subpopulation of human highly proliferative cells (hHiPCs) from adult epicardial tissue and found that they express ALK1, a high affinity receptor for bone morphogenetic protein-9 (BMP9), which signals via SMAD1/5 to regulate paracrine/autocrine signaling and angiogenesis. We show that in humans, circulating BMP9 level is negatively associated with the number of epicardial hHiPC and positively associated with endothelial cell (EC) number in the adult heart, implicating the potential importance of this signaling pathway in cardiac cell fate and vascular maintenance. To investigate BMP9/ALK1 signaling in hHiPCs, we selected a primary cell population of hHiPC from each of 3 individuals and studied their responses to BMP9 and BMP10 treatment *in vitro*. Proteins were collected in conditioned media (CM) for mass spectrometry and cell-based assays on human ECs and hHiPCs. Proteomic analysis of the hHiPC secretome following BMP9 or BMP10 treatment demonstrates that the secreted proteins, sclerostin (SOST), meflin/immunoglobulin superfamily containing leucine rich repeat (ISLR), and insulin-like growth factor binding protein-3 (IGFBP3), are novel regulated targets of BMP9/ALK1 signaling. Lentiviral shRNA and pharmacological inhibition of ALK1 in hHiPCs suppressed transcription and secretion of SOST, ISLR, and IGFBP3 following BMP9 treatment. Moreover, the BMP9-treated secretome of hHiPC increased capillary-like tube formation of ECs and hHiPCs. Treatment of hHiPCs with recombinant SOST increased *VEGF-a* expression, increased tube formation and enhanced expression of EC receptor marker annexin A2 (ANXA2). These data provide the first proteomic characterization of hHiPC, identifying BMP9/ALK1-mediated target protein secretion in hHiPCs, and underscore the complex role of BMP9/ALK1 signaling in paracrine/autocrine mediated angiogenesis. Data are available via ProteomeXchange with identifier PXD055302.

## Graphical Abstract

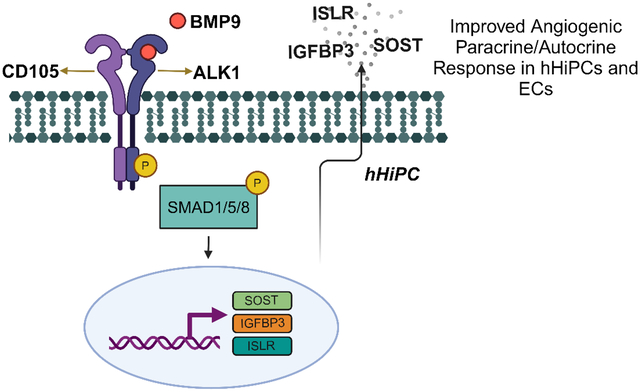

BMP9 binding stimulates ALK1 phosphorylation, SMAD1/5/8 phosphorylation, SOST/IGFBP3/ISLR transcription and paracrine secretion to improve whole secretome induced angiogenesis.

Created with BioRender.com

## Introduction

ALK1 (*ACVRL1*) serine/threonine receptor protein kinase signaling is a critical regulator of vascular development, regeneration, and myocardial repair after injury [1]. Bone morphogenetic proteins (BMP)-9 and -10 bind with high affinity to ALK1, which is prominently expressed in endothelial cells (ECs) [2,3], to activate downstream phosphorylation of SMAD1/5 transcription factors, and regulate key cellular processes such as cell proliferation, differentiation, and migration [3–5]. Studies have demonstrated that *Bmp9* is critical for myocardial repair after ischemic injury in mice, and conditional ablation of *Alk1* in endothelial cells results in adverse cardiac phenotypes [6–9]. Further, circulating BMP9 in humans is negatively associated with cardiovascular disease risk factors, suggesting the importance of this pathway in the adult myocardium [10,11]. In-depth understanding of the precise molecular mechanisms involved in BMP9/ALK1 signaling in cardiac cell-type-specific populations is necessary for the development of better therapeutics for the treatment of cardiovascular disease.

Angiogenesis is a critical mechanism involved in myocardial repair, and stimulating angiogenesis after injury may be an avenue for improving outcomes in patients with cardiac injury [12–14]. The formation of new blood vessels at the site of infarction can increase blood flow, enhance nutrient deposition, and decrease cardiomyocyte apoptosis [12]. This process can be mediated by paracrine and autocrine signaling of both endogenous and exogenous cell types and plays an important role in improving functional outcomes following cardiac injury [15,16]. We have recently demonstrated that the human adult myocardium harbors a subpopulation of highly proliferative cells (hHiPCs) that have the potential for therapeutic applications due to their highly proliferative discrete colony-forming ability and *ex vivo* expandability [17]. These cells express mesenchymal stem cell (MSC)/cardiac progenitor cell (CPC) markers CD105 (aka., the type III TGFβ/BMP co-receptor Endoglin, EGLN [18]), CD90, CD73, and CD29, with no expression of CD45, CD34, CD11b, or CD117 (c-kit) [17]. Further, hHiPCs have the capacity to differentiate toward an endothelial cell phenotype, harboring intrinsic pro-angiogenic properties *in vitro* [17,19–21].

Based on the prominent role of BMP9/ALK1 signaling in endothelial cells, we hypothesized that ALK1 signaling is critical for a pro-angiogenic paracrine/autocrine response in EC and hHiPC. We found that ALK1 is expressed in hHiPC and identified novel BMP9-target secreted proteins using unbiased global proteomics analysis of the hHiPC secretome *in vitro*. Lentiviral knockdown and pharmacological inhibition of *ALK1*/ALK1 were used to confirm novel regulatory targets in this pathway for this cell-type specific population. Corresponding regulated targets such as sclerostin (SOST) were assessed for involvement in endothelial cell morphogenesis *in vitro* supporting the hypothesis that the BMP9 secretome and its constituents are potential critical drivers of this response.

## Materials and Methods

### Human subjects

The research was performed in accordance with study protocols approved by Maine Medical Center Institutional Review Board, which is accredited by the Association for the Accreditation of Human Research Protection Programs (AAHRPP). The study cohort consisted adult subjects recruited to undergo intra-operative myocardial biopsy at the time of scheduled coronary artery bypass grafting surgery at Maine Medical Center (MMC) in Portland, Maine. Subjects 18 years of age or older were approached for this study, and all subjects provided informed consent. Exclusion criteria included the presence of active myocarditis, hypertrophic cardiomyopathy, constrictive pericarditis, significant valvular and/or pericardial disease, severe pulmonary hypertension, significant hepatic disease, renal impairment (creatinine > 2.5 mg/dL), severe ventricular arrhythmias, malignancy other than non-melanoma skin cancers, expected survival less than one year and inability to provide informed consent. Patient demographic information is highlighted in Table 1.

### Cell culture, shRNA transfection, and *in vitro* stimulation

hHiPCs were isolated according to previous methods [17,19]. In brief, minced left ventricle (LV) epicardial tissue was incubated in digestion solution (10 mg/ml collagenase II, 2.5 U/ml dispase II, 1 μg/ml DNase I, and 2.5 mM CaCl_2_, Sigma, Saint Louis, MO) for 45 minutes at 37°C. Prior to colony formation, cells were depleted of myocytes by passage through a 70-μm cell strainer and sorted through magnetic separation of CD45^+^ immune cells. The resulting CD45^−^ cells were plated in single-cell suspensions on 48-well plates at a cell density of 500 cells per cm^2^ and formed colonies with high proliferation rates compared to non-colony-forming cells [21]. hHiPCs were grown in 10 cm CytoOne plates in M199-EGM2 (3:1, v/v) with 10% FBS and 1% antibiotic-antimycotic solution (Sigma, St. Louis, MO). Experiments were conducted in triplicate at passages 4–6. Primary human retinal endothelial cells (HRECs, Cell Systems, Kirkland, WA) or human dermal microvascular endothelial cells (HMEC-1, ATCC, Manassas, VA) were cultured in EGM2 media at 37°C in 5% CO_2_ and used under passage 10. For viral transduction, hHiPCs were treated with 8 μg/mL polybrene (MISSION^®^, La Jolla, CA) and sh*ALK1* or non-targeting (shNT) particles (MISSION^®^, La Jolla, CA) with a MOI=1. Puromycin (1 ug/mL, Gibco^™^) selection was performed for 48 hours. Afterwards, confluent cells were treated with BMP9 (5 ng/mL, R&D Systems, Minneapolis, MN), with BMP10 (10 ng/mL, R&D Systems, Minneapolis, MN), or vehicle control (0.1% BSA) in low serum/phenol red-free media (1% FBS) for 24 hours. These concentrations were chosen relevant to physiological levels in the blood and for correlation in the plateau of SMAD1/5 upregulation [3,22,23]. For sclerostin (100 ng/mL, R&D Systems, Minneapolis, MN) or vehicle control (0.1% BSA) treatment, hHiPCs were treated in low serum media for 24 hours. This concentration was chosen due to potential of SOST as an angiogenic factor as described previously [24].

### Flow cytometry

Methods were conducted as described previously [17]. In brief, hHiPCs were treated with Cytofix/Cytoperm^™^ (BD Biosciences, Franklin Lakes, NJ, Cat# AB-2869008) to fix and permeabilize cells. Cell-surface antigen expression was examined using the following antibodies: FITC-conjugated CD45 (HI30) and APC/Cy7-conjugated CD31 (WM59) at 1:200 dilution (all from BioLegend, San Diego, CA).

Intracellular antigen expression was assessed using the following antibodies: anti-ALK1 human (Proteintech Group, Rosemont, IL, Cat# 14745-1-AP) at 1:200 dilution, goat anti-rabbit IgG Alexa Fluor 488-conjugated (Abcam, Carlsbad, CA, Cat# ab150077) at 1:500 dilution, IgG isotype control anti-rabbit (Abcam, Carlsbad, CA, Cat# ab172730) at 1:200 dilution, or unstained (without primary antibody or secondary antibody). Data acquisition was performed on the MacsQuant Analyzer 10 (Miltenyi Biotec Inc., Auburn, CA) and analyzed using FlowJo (10.10). Delta mean fluorescent intensity (MFI) was calculated as MFI of cells stained with the specific anti-ALK1 antibody minus MFI corresponding to the isotype and unstained control cells.

### Protein preparation

Protein preparation was conducted as described previously [22]. Briefly, hHiPCs were treated in low serum/phenol red-free media for 24 hours with BMP9 (5 ng/mL) or vehicle control. Conditioned media (CM), was collected 24-hours following treatment, spun at 500 × g for 10 minutes to remove cellular debris, and concentrated using a 5 kDa molecular weight cutoff (MWCO) ultrafiltration device (Agilent, Santa Clara, CA, Cat# 5185-5991). Protein concentrations were measured by the BCA protein assay (Pierce, Rockford, IL, Cat# 23225). CM protein (100 μg) was precipitated using 9 volumes of ice-cold ethanol and frozen at −80°C overnight. Proteins were pelleted (4°C for 20 minutes at 15,000xg) and denatured using 8 M Urea/50 mM Tris-HCL (pH=8.0). Proteins were reduced using 25 mM dithiothreitol (DTT) and alkylated using 15 mM iodoacetamide. Samples were diluted to below 1 M urea with 50 mM Tris-HCL (pH=8.0). The sample were then digested using 2.5 μg of sequencing-grade trypsin (Promega, Madison, WI, Cat# V5111) overnight at 37°C. Digested peptides were purified using Pierce C18 spin columns, dried, and stored at −20°C before reconstitution in 2% acetonitrile (ACN), 5% formic acid followed by LC-MS/MS analysis. For cell lysates, cells were dissociated using trypsin, washed with ice-cold PBS, and lysed using 8M Urea, 50 mM Tris-HCl pH 8.0, and 1 mM DTT. Alkylation was performed at room temperature in the dark with iodoacetamide at a final concentration of 10 mM. Trypsin digestion and peptide clean-up were performed as described for CM.

### LC-MS/MS

For unbiased protein identification and ion library generation, we used a data dependent acquisition (DDA) workflow, while **s**equential **w**indow **a**cquisition of all **th**eoretical mass spectra (SWATH) was employed for relative protein quantification in all samples [25]. Nano-LC was performed on an UltiMate 3000 RSLCnano (Thermo Fisher, Waltham, MA). The separation column was fabricated in-house (ReproSil-Pur C18-AQ, 5 μm particle, 120 Å pore, Dr. Maisch GmbH, Ammerbuch, Germany; Column dimensions 50 μm I.D. × 15 cm length). Gradients were performed with Burdick & Jackson LC-MS-grade solvents (Honeywell, Morris Plains, NJ) with Optima-grade formic acid (Fisher Chemical, Pittsburgh, PA). Channel A pumped 0.1% formic acid, while channel B ran 0.1% formic acid in ACN. The separation column was equilibrated at 45°C with 4% B at 300 nL/min. Flow rate was increased to 350 nL/min for sample loading. In all cases, approximately 1 μg of sample was loaded via the autosampler fitted with a 3.6 μL silica capillary sample loop. After 10 minutes of loading, the loop was taken out of the flow path and flow rate returned to 300 nL/min. A linear gradient to 30% B at 95 minutes was then executed, followed by a gradient to 50% B at 113 minutes, then a rapid gradient to 92% B at 115 minutes. Washing was performed to a run time of 120 minutes, at which point the system was returned to starting conditions and equilibration performed for 10 minutes before the next sample was run. Column eluate was directed through a stainless-steel union into the mass spectrometer via a silica emitter (10 μM I.D. PicoTip, New Objective, Littleton, MA), which interfaced the mass spectrometer (TripleTOF 5600, Sciex, Framingham, MA) via a Nanospray III source operating at 2600 V and 150°C employing a nitrogen sheath gas at 14 psi and curtain gas running at 23 L/min.

To generate spectral libraries for subsequent SWATH and quantitative analyses, DDA MS experiments were performed in high-sensitivity mode utilizing a MS parent ion scan from 400–1250 amu and an accumulation time of 250 msec. Criteria required for candidate selection in the MS/MS experiments were a minimum target peak intensity of 350 counts per second (cps), a charge state of 2–5, and selection of 50 candidate ions per cycle. After MS/MS analysis, also in high-sensitivity mode, candidate parent ions (50 mDa mass tolerance) were excluded from subsequent selection and sequencing for 12 seconds. MS/MS spectra were acquired from 100–1250 amu using an accumulation time of 20 msec and rolling collision energies determined via parameters defined by the instrument manufacturer. A collision energy spread was not used. These experiments were used to generate spectral libraries for subsequent SWATH and quantitative analyses.

Peptide quantification was performed by data-independent analysis (DIA, SWATH) on the same instrumentation as used for the DDAs, essentially as previously reported [26,27]. While chromatographic and mass spectrometer source conditions were identical to those used in DDA mode, SWATH analysis was started with a parent ion scan in high-sensitivity mode employing an accumulation time of 50 milliseconds (ms). Each of 100 mass windows used for subsequent analyses was approximately 6 mass units wide and overlapped by 1 mass unit. Collision energies selected were for doubly charged ions of the corresponding mass window, and a 5 V collision energy spread was used in all cases. Accumulation time was set to 96 ms for each window. All SWATH experiments were performed in triplicate.

For multiple reaction monitoring (MRM) of specific secretome proteins identified by SWATH analysis, peptides prepared as above were subjected to LC/MS and MS/MS fragments (i.e., Q1 and Q3 transmission, respectively) on a Sciex 6500^+^ triple quadrupole mass spectrometer [28]. Candidate peptide parent (Q1) and product (Q3) ions for each protein were obtained using the human MRM library build on the SRMAtlas utility [29]. Peptides were separated on a reverse-phase column fabricated in-house (ReproSil-Pur C18-AQ, 5 μm particle, 120 Å pore, Dr. Maisch GmbH; Column dimensions 150 μm I.D. × 15 cm length) operating at a flow rate of 1.7 μL / minute. MRM experiments were conducted in positive ion mode using the following values for Q1/Q3 transition acquisition: ISV = 4800V, DP = 55V, CXP = 15V, and a floating collision energy was determined as recommended by SRMAtlas [29,30].

### Mass spectrometry data analysis

Protein identification for ion library generation was determined for six representative DDA runs using the Paragon algorithm [31] in the ProteinPilot software package (Sciex, Version 5.0.2) searching a Uniprot reviewed human database with a <5% false discovery rate (FDR) at the peptide level.

Quantification data was generated using the SWATH Acquisition MicroApp (version 2.0.1) within the Sciex PeakView software (version 2.2.0). A maximum of 6 peptides per protein were used for quantification with each analyte being characterized by a maximum of 6 transitions. Each peptide peak was extracted over a 15-minute window at a resolution of 75 parts per million (ppm). A peptide confidence threshold of at least 95% was required, as was a false discovery rate threshold of 5%.

Relative protein expression levels from both SWATH and MRM data were determined using MarkerView [32] (Sciex) and data were subjected to most-likely ratio (MLR) normalization [33] for SWATH. Total area sums (TAS) normalization was applied to MRM data, as described previously [34]. Background was determined by instrument detection limit (IDL) as described [35]. P-values for fold-change were calculated using Welch’s t-test in MarkerView. The mass spectrometry data have been deposited with the ProteomeXchange Consortium via the PRIDE [36] partner repository with the dataset identifier, PXD055302.

### RNA extraction, cDNA preparation and real-time PCR (RT-qPCR) analysis

Total RNA from hHiPCs and HRECs was collected and purified using the Rneasy kit (Qiagen). mRNA was converted to cDNA using the iScript^™^ cDNA Synthesis Kit (Biorad, Hercules, CA, Cat#: 1708890). RNA was quantified using SYBR green PerfeCTa SYBR Green SuperMix (Quantabio, Beverly, MA, Cat#: 95054-500) and gene primers (NCBI Blast). The expression of human target genes (*ALK1, IGFBP3, ISLR, CD105, SOST*, and *CXCL5*) were analyzed using real-time quantitative PCR (RT-qPCR, CFX Connect 384, Biorad, Hercules, California) and CFX Manager 3.1 software. *CD105* was used as a positive control. RNA expression levels were normalized to the validated housekeeping gene *18S*. Amplification primer specificity was assessed using melt curve analysis (CFX Manager) where product melting temperature was determined with negative first derivative of relative fluorescence units versus the temperature (-dRFU/dT). Quality assessment, differential expression using the ΔΔCT method, and t-tests were performed using R package ‘pcr’ [37]. Validated forward and reverse primers are listed in Table 2.

### Tube formation

To investigate changes related to *ALK1* knockdown and BMP9 treatment, vehicle and BMP9-treated CM was incubated with human retinal endothelial cells (HREC, Lonza) and hHiPCs to determine the direct effect of changes in secreted proteins on tube formation. HRECs or hHiPCs (75,000 cells) were plated in 24-well plates coated with Matrigel (Corning, New York, NY) at a concentration of 10 mg/mL. Cells were treated with either BMP9 (5 ng/mL), SOST (100 ng/mL), hHiPC BMP9-CM (300 ug/mL added, based on total protein, as measured above), or vehicle-CM (300 ug/mL) for 6-hours. HREC or hHiPC tube formation was captured using a phase contrast microscope Olympus IX-70 and analyzed using the “Angiogenesis Analyzer” plugin in ImageJ [38,39].

### Statistical analysis

Normality testing was calculated using PRISM with normal (Gaussian) distribution. This was already included above. Principle component analysis (PCA) of data was conducted in MarkerView as previously described [32,40]. Data set group comparisons were analyzed using unequal variance (Welch’s) t-test unless otherwise indicated [32]. A p-value of less than 0.05 was considered significant.

## Results

### Characterization of mesenchymal cell markers in hHiPC

**Figure 1.**
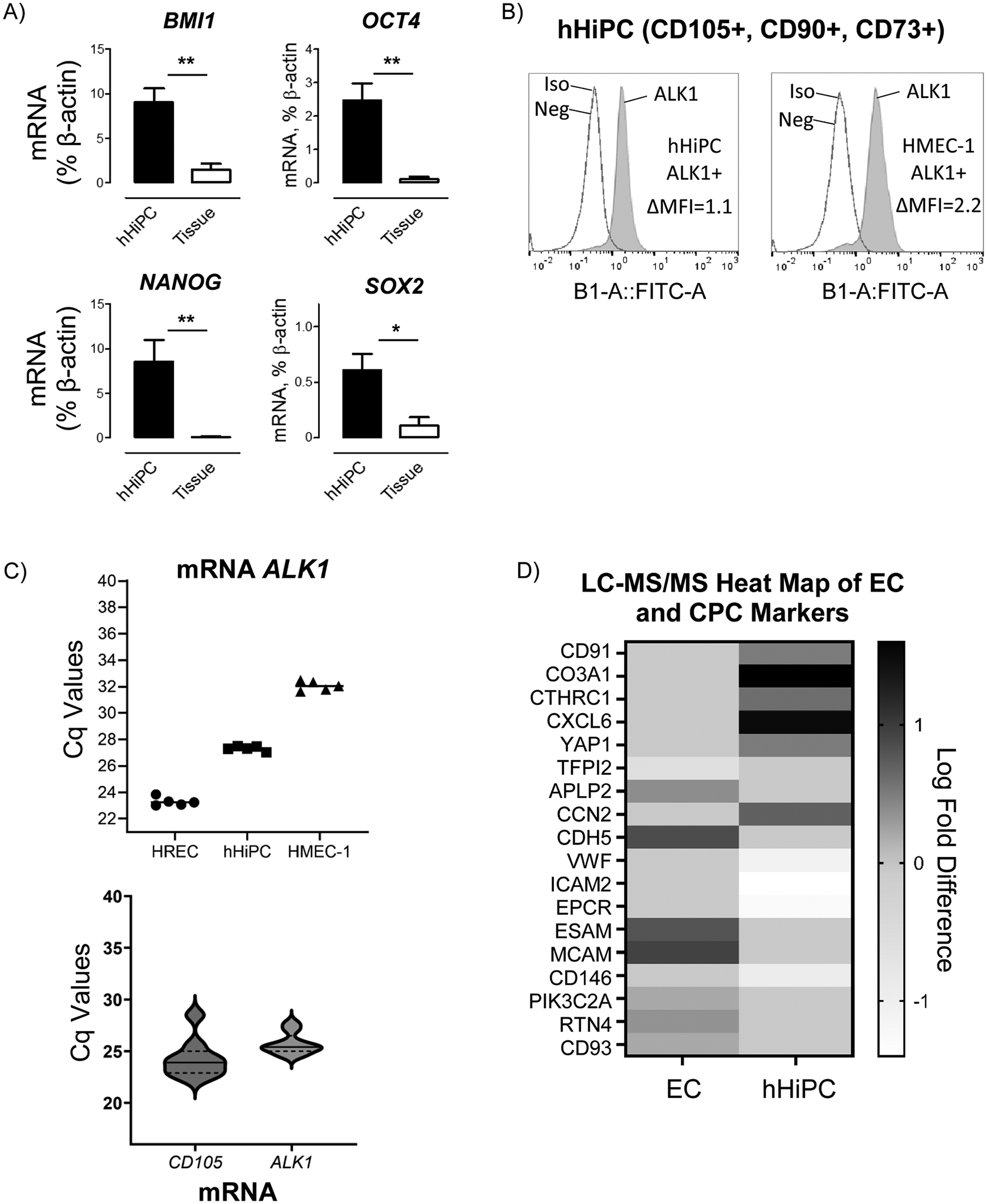
hHiPCs express stem and progenitor cell markers and surface expression of the BMP9 receptor ALK1. **A)** Quantitative RT-qPCR analysis of mesenchymal/progenitor marker expression in hHiPC compared to whole ventricular tissue (n=3 distinct patient-derived clones, measured in triplicate) normalized to β-actin; P-value by unpaired t-test (** indicates p<0.01, * p<0.05). **B)** hHiPC and HMEC-1 (positive control) ALK1 protein levels were analyzed using flow cytometry. Expression of ALK1 (grey) compared to combined IgG isotype control (Iso) and non-stained (Neg). Data presented as delta MFI (n=2 distinct patient-derived clones, measured in triplicate). **C)** Upper Panel; RT-qPCR of *ALK1* mRNA (n=5 distinct patient-derived clones, measured in triplicate) in primary endothelial cells in human retinal (circles), human microvascular (triangles) endothelial cells (HREC, and HMEC-1, respectively), and hHiPC (squares). Lower panel: hHiPC clones (n=9 distinct patient-derived clones, measured in triplicate) were surveyed for levels of *CD105* and *ALK1* by RT-qPCR. Plots indicate mean Cq (solid lines) and 1 standard deviation from the mean (dashed lines). **D)** SWATH LC-MS/MS analysis of normalized counts of top differentially expressed mesenchymal and EC protein markers in hHiPC (n=9) and EC (n=4) lysates. P-value by Welch’s t-test.

hHiPCs have been previously defined as expressing mesenchymal stem cell markers related to the TGFβ signaling pathway, such as CD105 [17,20]. We sought to further characterize hHiPCs using RNA and protein analysis. Using RT-qPCR analysis, we found that hHiPCs highly express transcription factors of mesenchymal stem cells; *BMI1*, *OCT4*, *NANOG*, and *SOX2* compared to whole ventricular tissue expression (Figure 1A), supporting the mesenchymal-like classification of these cells. We next wanted to establish whether the high-affinity receptor for BMP9 and co-receptor for CD105, activin A receptor-like type 1 (ALK1), is also expressed in isolated hHiPCs. Using flow cytometric analysis, we found ALK1 protein expression in both hHiPCs and human microvascular endothelial cells (HMEC-1) as previously described as high-expressing ALK1 cells (Figure 1B) [22,41].

RT-qPCR was used to estimate the relative levels of *ALK1* mRNA in hHiPC and two primary endothelial cell populations, human retinal and human microvascular endothelial cells (Figure 1C, upper panel). This analysis indicated that hHiPC levels of *ALK1* mRNA were robust and within the range expected for endothelial cells, which are known to express ALK1 [42,43]. Next, we estimated the degree of variation of *ALK1* expression for hHiPC clones isolated from bypass graft surgery patients (Figure 1C, lower panel, n=9). Also shown are data for *CD105*, a phenotypic marker for hHiPC [17,44]. *ALK1* and *CD105* mRNA levels were variable across the clones measured, but all clones had substantial levels of *ALK1*, suggesting that most or all *CD105*^+^ hHiPC isolates were also *ALK1*^+^.

To further investigate the potential of hHiPCs as a pre-endothelial mesenchymal cell type, we used unbiased SWATH proteomic analysis to assess protein expression levels in hHiPCs, using a more-differentiated vascular endothelial cell-type as a reference. Compared to cultured human retinal endothelial cells (HREC), hHiPCs expressed a 35.4-fold higher level of CXCL6 protein, which is a novel characteristic marker of cardiospheres [45], and a 3-fold higher YAP1 protein expression level, which is an essential transcriptional activator in stem cells (Figure 1D) [46–48]. hHiPCs also exhibited lower expression of EC surface markers such as ICAM2, EPCR, VWF, CDH5, and CD146 and higher expression of mesenchymal cell markers CCN2, CD91, and CTHRC1 (Figure 1D), further supporting the progenitor-like nature of these cells, and expand upon results of previous studies that hHiPCs isolated from cardiac tissue of coronary artery bypass graft (CABG) patients are highly proliferative cardiac progenitor-like cells that express mesenchymal markers, such as CXCL6, as well as the BMP9 receptor ALK1.

### Molecular characterization of the hHiPC secretome

**Figure 2.**
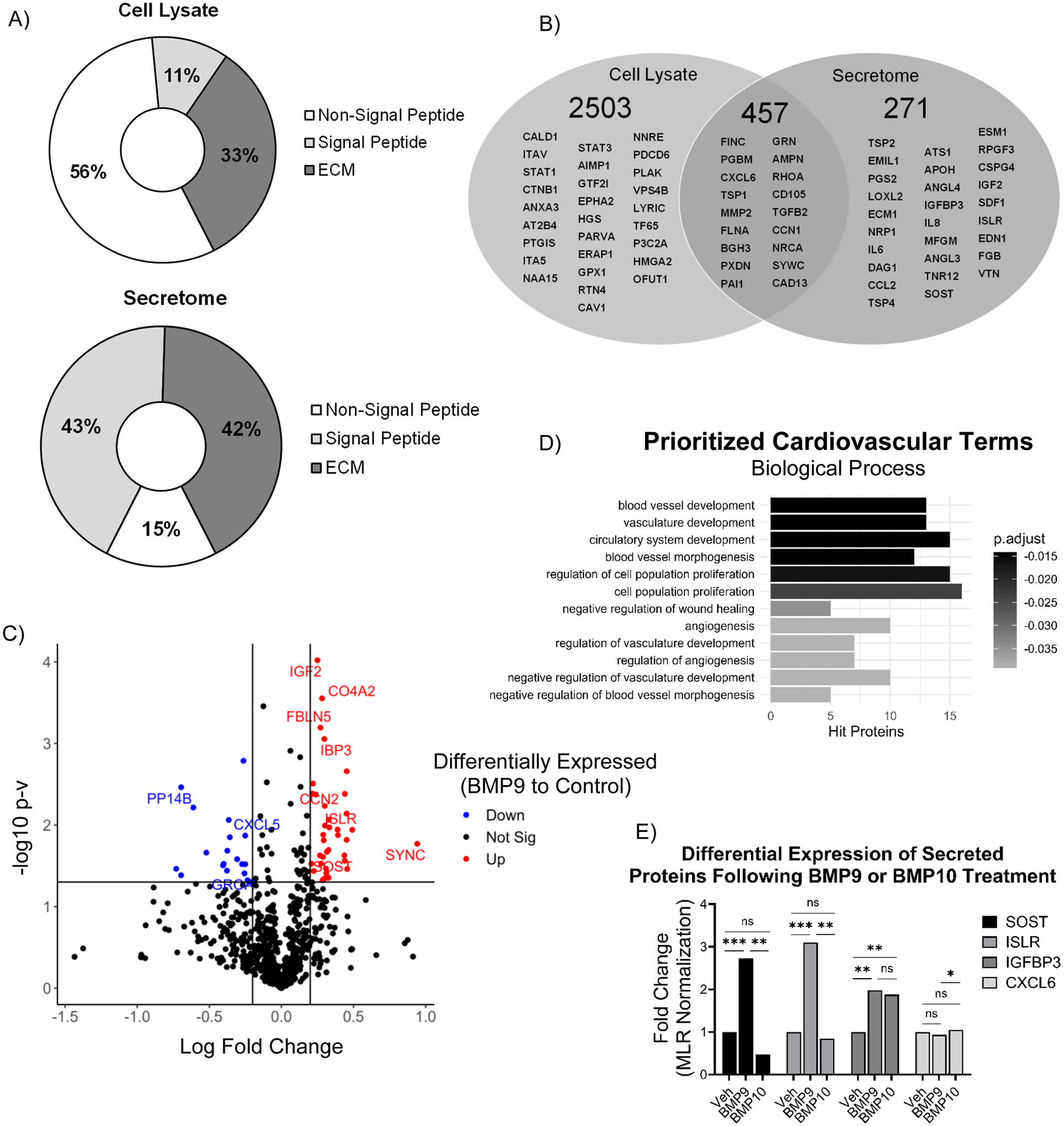
Characterization of the BMP9-treated hHiPC secretome. Liquid chromatography/mass spectrometric analysis of BMP9-treated hHiPC. **A)** Pie chart of detected signal sequences and ECM ontology compared to non-signal sequence proteins determined by LC-MS/MS SWATH with FDR analysis (n=3). **B)** Venn diagrams illustrating secretome and cell lysate proteins detected by LC-MS/MS. **C)** Volcano plot of differentially BMP9-regulated secretory proteins (p<0.05, fold-change>1.5, < 0.6). Red indicates significantly higher with BMP9 treatment, and blue indicates significantly lower in the vehicle control group. **D)** Gene ontology enrichment analysis of differentially upregulated proteins. Upregulated secreted proteins were classified as p<0.05 and fold-change >1.6. **E)** SWATH LC-MS/MS analysis of BMP9 or BMP10 CM prioritized targets SOST, ISLR, IGFBP3, and CXCL6. Significance was determined by Welch’s t-test. Data are expressed as normalized fold-change (*** indicates p<0.001, ** p<0.01, * p<0.05. ‘ns’ p>0.05).

We applied DDA/SWATH to identify and quantify proteins with altered expression levels in BMP9 or BMP10-treated hHiPCs as compared to controls. We prepared total cell lysates and low-serum conditioned media (CM) from cultured hHiPCs. SWATH LC-MS/MS analysis of the CM suggested a substantial enrichment of secreted (43%) or extracellular matrix (ECM, 42%) proteins, based on those characterized in the UniProt database, compared to total cell lysate analysis of detected secreted (11%) and ECM (33%) proteins (Figure 2A). LC-MS/MS DDA confirmed pro-angiogenic and pro-regenerative proteins that were detected in both the hHiPC lysates and secreted protein fractions (Figure 2B), such as CD105 and CXCL6. Known BMP-regulated proteins, for example plakoglobin (PLAK, *JUP*) [49,50] and caveolin-1 (CAV1) [51,52], were detected in total cell lysates, but not in the secretome (Figure 2B). In the hHiPC secretome, LC-MS/MS analysis identified IGFBP3, IGF2, SDF1 (CXCL12), SOST, and ISLR, but these proteins were not identified in the cell lysate proteome (Figure 2B). These comparison studies between the total cell proteome and secretome highlight the need for secretome-specific analysis rather than studying the total cell lysate proteome or secretome alone to gain greater identification of secreted proteins.

DDA of CM from BMP9 or BMP10-treated and control hHiPCs resulted in a total of 878 protein identifications, which provided the ion library for subsequent quantitative SWATH analyses. Differentially-expressed proteins were defined as having p-values<0.05 and log fold-changes>0.2 for upregulated proteins and <−0.2 for downregulated proteins (Figure 2C). The top 25 differentially-expressed (BMP9-treated versus control) proteins are listed in Table 3. Prominent upregulated proteins following treatment with BMP9 include sclerostin (SOST), insulin-like growth factor binding protein 3 (IGFBP3) and meflin (ISLR, Figure 2C). Gene enrichment analysis was performed using clusterProfiler (RStudio) and terms were prioritized according to cardiovascular gene ontology annotation (Figure 2D) [14]. Enrichment scores identified high enrichment of upregulated proteins for biological processes including angiogenesis, blood vessel development, cell proliferation, and vasculature development. No terms were enriched for downregulated proteins, which potentially reflects a lower differential expression of downregulated secretory proteins (number of proteins=19) compared to upregulated proteins (number of proteins=40). However, it is important to note that the downregulated secretory proteins CXCL5 and CXCL1 (GROA) are important regulatory cytokines known to be involved in several angiogenic/inflammatory pathways [53,54]. To understand the potential for ligand-dependent differences in ALK1 signaling, BMP9 upregulated target proteins were compared to BMP10 treatment (Figure 2E). SOST and ISLR were significantly different in BMP9 treatment compared to BMP10. IGFBP3 was significantly different compared to controls, but not significantly different between treatments. As expected, CXCL6 protein expression was not changed in either treatment compared to control. Together, these data identify novel characterization of BMP9-specific paracrine and autocrine signaling targets in hHiPC.

### Transcriptional analysis of putative BMP9-regulated hHiPC secreted proteins

**Figure 3.**
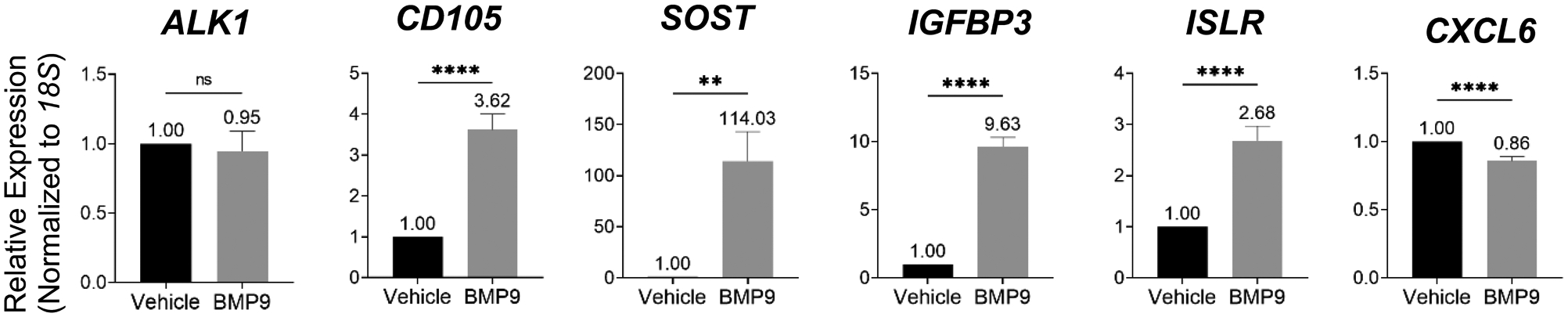
Transcriptional regulation of secretome proteins by BMP9. **A)** RT-qPCR of hHiPC (n=5 independent patient-derived clones triplicate measurements). cDNA was used to assess BMP9-dependent RNA expression for *SOST, IGFBP3, ISLR*, and *CXCL5*. RT-qPCR responses were normalized to *18S*. Data were analyzed by student’s t-test and presented as mean fold-change (pooled per individual clone) +/− SD. *** indicates p<0.001, ** p<0.01, * p<0.05, ‘ns’ p>0.05.

To corroborate selected LC-MS/MS data with analysis of BMP9 target secreted proteins we used RT-qPCR analysis to determine transcriptional regulation of interesting proteins identified in the secretome analysis. Following BMP9 treatment in hHiPC, *ALK1* mRNA levels remained unchanged, as expected. We found a significant increase in the mRNA levels of the known BMP9-target *CD105* (positive control) [55]. RT-qPCR measurements also confirmed upregulation of mRNA levels for *SOST*, *IGFBP3*, and *ISLR*, and decreased expression of *CXCL5*, although fold-change expression of mRNA *CXCL5* is low (−0.86) compared to fold-change expression of secreted CXCL5 (−0.55) (Figure 3). These data further support the finding that SOST, IGFBP3, ISLR, and CXCL5 are regulated targets of BMP9.

### The BMP9-treated secretome increases endothelial cell tube formation

**Figure 4.**
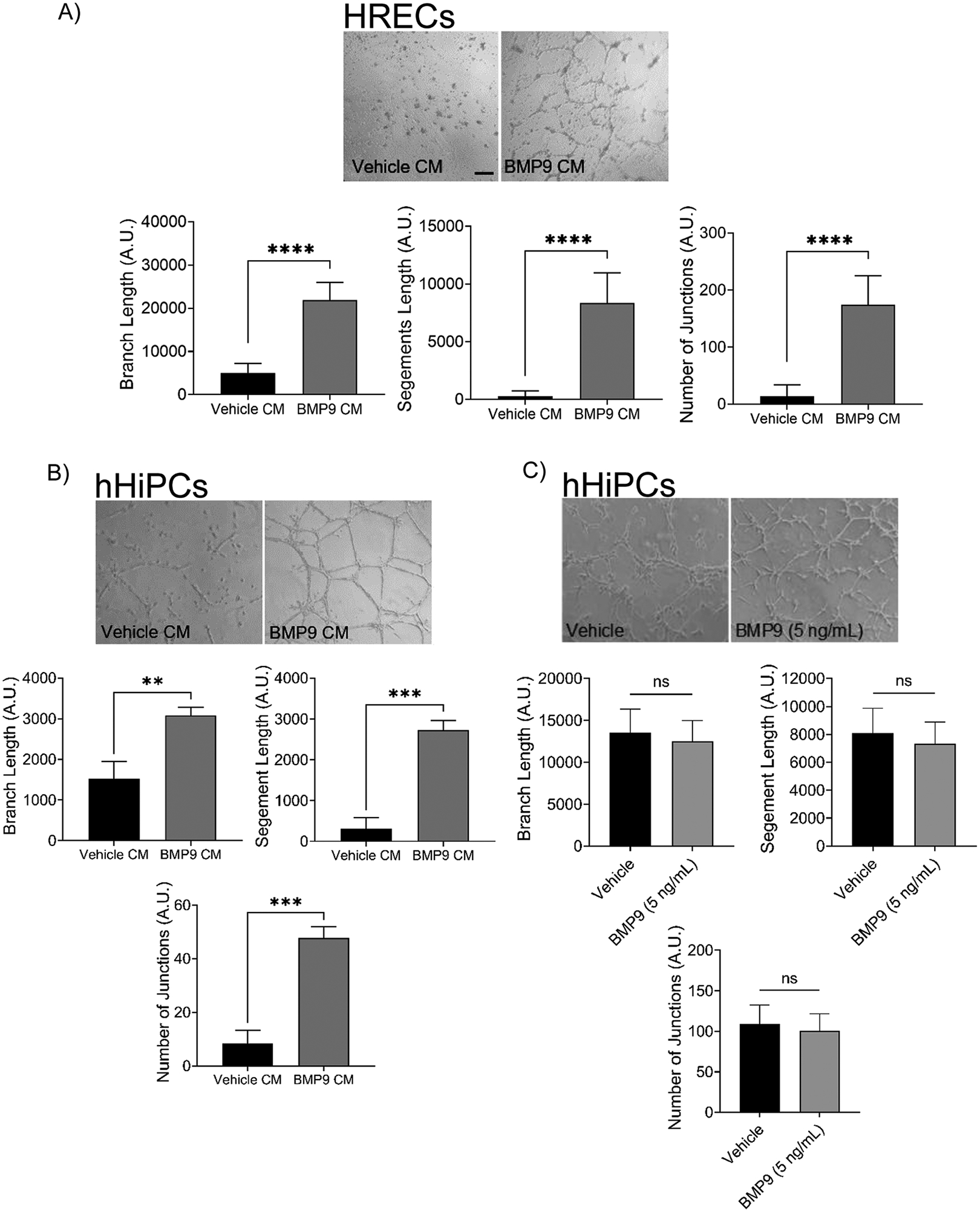

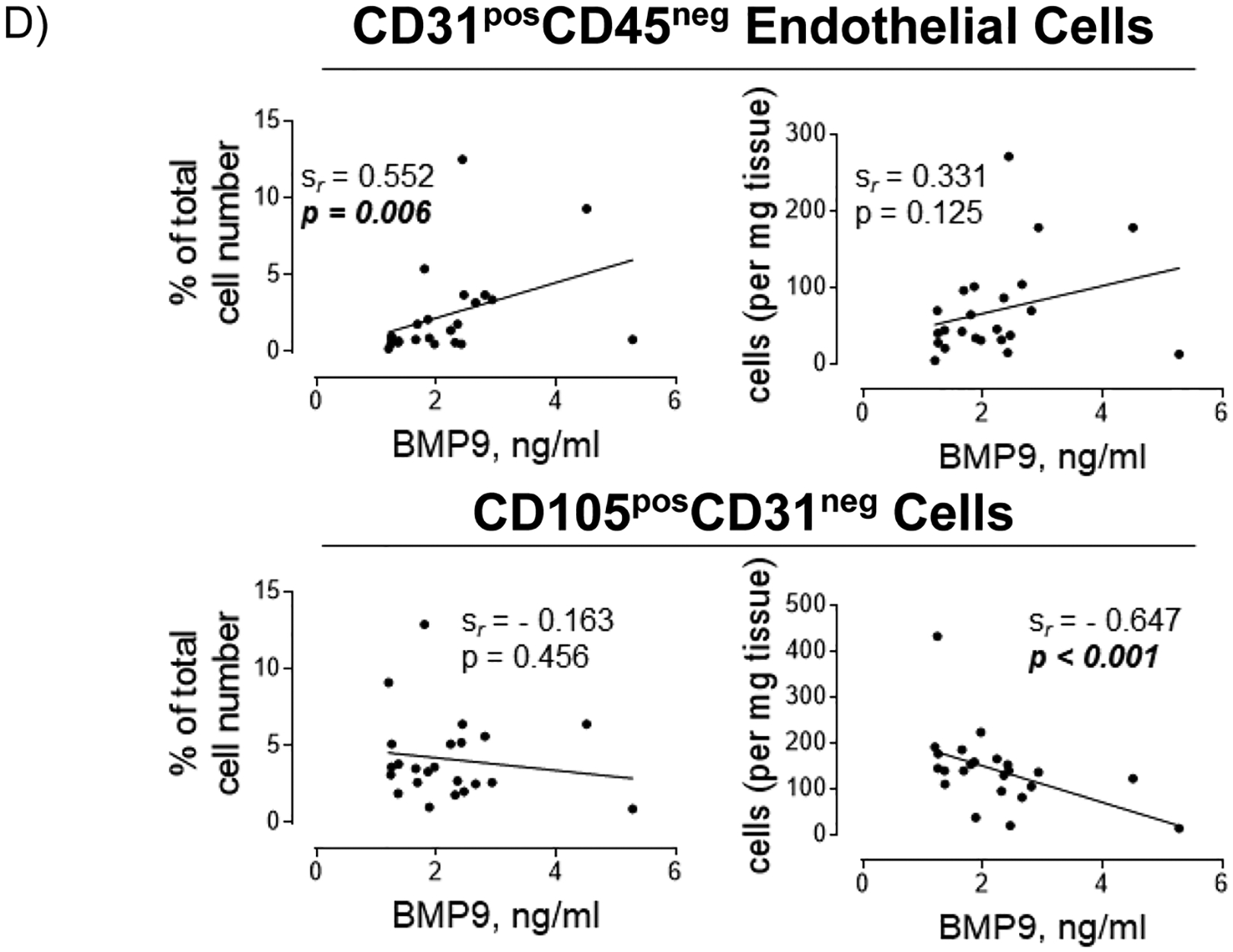
Conditioned media (CM) from BMP9-treated hHiPCs and HRECs improves tube formation *in vitro*. Representative cell culture images and tube formation parameter measurements for: **A)** HREC (n=3 biological replicates measured in technical triplicate) and **B)** hHiPC (n=3 biological replicates from one patient derived clone measured in technical triplicate), following incubation with hHiPC BMP9 CM compared to vehicle treated hHiPC CM. **C)** To assess potential direct BMP9-dependent effects on tube formation, hHiPCs (n=3) were plated on Matrigel (Corning) in low serum M199 with vehicle control or BMP9 (5 ng/mL). **(A-C)** Total branch length, segment length, and junction number were measured using the Angiogenesis Analyzer in ImageJ. Data are expressed as mean ± SD; P-value by unpaired t-test (**** indicates p<0.0001, *** p<0.001, ** p<0.01, ‘ns’ p>0.05). Scale bar, 100 μm. **D)** Flow cytometric analysis of CD31^pos^CD45^neg^ EC and CD105^pos^CD31^neg^ hHiPC was performed immediately after preparation of cell suspensions from LV biopsies (n=23). Levels of BMP9 in plasma were measured using ELISA. Spearman correlation coefficients and p-values are as indicated.

To determine the phenotypic role of the hHiPC BMP9 treated secretome *in vitro*, we used the Matrigel tube formation assay to determine the direct effect of BMP9-treated CM on both hHiPC and HRECs. We observed significantly improved tube formation in both hHiPC and HREC treated groups suggesting a potential pro-angiogenic autocrine and paracrine role of the BMP9 treated secretome (Figures 4A and 4B). We further determined that independent treatment of hHiPC with BMP9 alone did not enhance tube formation (Figure 4C), as compared to BMP9-treated CM (Figure 4B), further supporting hHiPC BMP9 target factors as major drivers of patient derived hHiPC and HREC tube formation. With the prominent role of BMP9/ALK1 signaling in the human heart and vasculature, we also sought to determine the relationship between circulating BMP9 in CABG patients and number of biopsied hHiPC (CD31^−^, CD105^+^) and EC (CD31^+^, CD45^−^). We found a negative correlation (p<0.001) between circulating BMP9 levels and the number of hHiPCs, and a positive correlation in percentage of ECs (p<0.006) suggesting a potential role for BMP9 signaling in hHiPC function through promotion of differentiation into endothelial cells (Figure 4D). These results further illustrate the potential *in vivo* role of BMP9 signaling in hHiPC through promotion of paracrine/autocrine signaling and endothelial cell morphogenesis.

### hHiPCs require ALK1 for BMP9 regulation of IGFBP3, SOST, and ISLR

**Figure 5.**
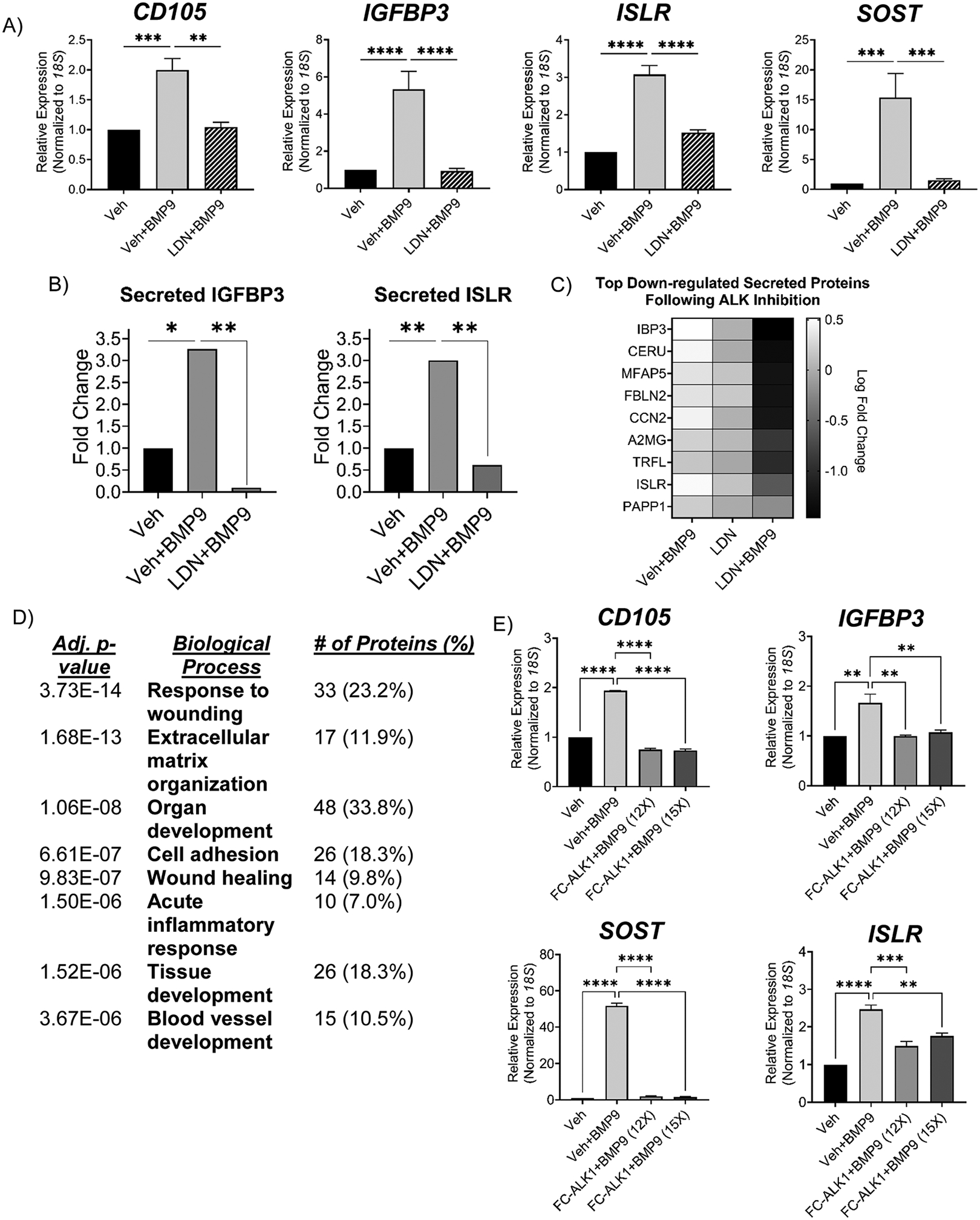

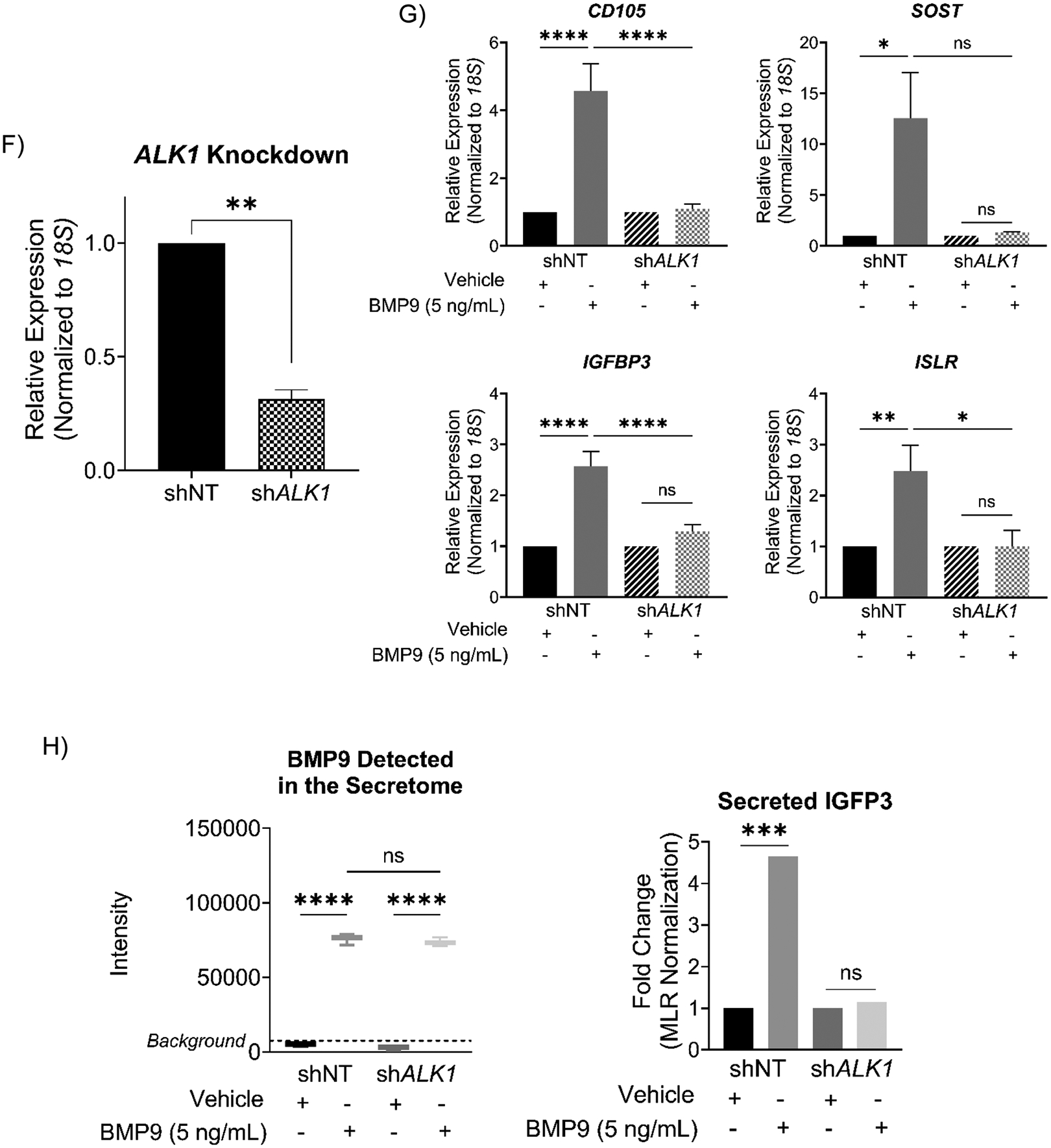
hHiPCs require ALK1 for angiogenic secretome response via BMP9. **A)** Analysis of treatment targets (n=3 independent patient-derived clones measured in technical triplicate). mRNA measurements were analyzed by student’s t-test. Data were normalized to *18S*. Data are expressed as fold-change ± SD relative to vehicle (veh) treated control. **B, C)** LC-MS/MS SWATH quantitative analysis of treated hHiPC secretome targets proteins (n=3). Heat map (FC>1.6, p-value<0.05) and normalized intensities were calculated using MLR normalization. Data are presented as mean fold-change relative to control. **D)** Enrichment analysis of biological process and cellular component using LC-MS/MS secretome analysis following ALK1 inhibition. **E)** mRNA analysis of BMP9 targets following BMP9 and Fc-ALK1 treatment at 12X and 15X molar excess (n=3). Data were normalized to *18S*. Data are expressed as relative fold-change ± SD (‘ns’ indicates p>0.05, * p<0.05, ** p<0.01, *** p<0.001). **F, G)** mRNA measurements following lentiviral knockdown of *ALK1* and BMP9 treatments were taken in triplicate and analyzed by unpaired t-test (n=4 independent patient-derived clones measured in technical triplicate). Data were normalized to *18S*. Data were analyzed by Student’s t-test and presented as mean fold-change ± SD (**** indicates p<0.0001, ** p<0.01, * p<0.05, ‘ns’ p>0.05). **H)** LC-MS/MS analysis with SWATH acquisition was used to test for levels of BMP9 from both endogenous expression compared to background (dashed line) and following addition of human recombinant BMP9 (left panel). SWATH LC-MS/MS analysis of IGFBP3 with and without shALK1-mediated suppression of ALK1 (n=3). Data shown are following MLR normalization. Significance was determined by Welch’s t-test. Data are expressed as normalized fold-change (*** indicates p<0.001, ‘ns’ p>0.05).

We first used pharmacologic inhibition of ALK1 to elucidate the potential requirement for ALK1 in hHiPC BMP9 responsiveness and secretion. It is important to note that fully ALK1-specific small-molecule inhibitors are not available: LDN-193189 (LDN) inhibits ALK1 (IC50 of 0.8 nM) as well as ALK2, 3, and 6 (IC50 of 0.8 nM, 5.3 nM, and 16.7 nM respectively) [57]. Nonetheless, we sought to establish the feasibility of inhibiting this pathway for regulatory target repression. Upon treatment with BMP9 for 24 hours, hHiPC cells demonstrated significant increases in transcription of CD105 (positive control), IGFBP3, ISLR, and SOST genes as measured by mRNA levels (Figure 5A, Veh+BMP9 vs. Veh). Pretreatment of the cultures for 30 minutes with 0.5 μM LDN abrogated the induction of these mRNA expression levels relative to those seen with BMP9 treatment alone (Figure 5A, Veh+BMP9 vs. LDN+BMP9), indicating that under the conditions tested, LDN treatment represses BMP9-dependent induction of these targets.

We used variable window SWATH LC-MS/MS [27,58] of CM to measure changes in the BMP9-treated secretome following ALK1 inhibition and confirmed decreased secretion of IGFBP3 and ISLR following treatment with LDN (Figure 5B). Unbiased LC-MS/MS analysis indicated differential regulation of other secreted proteins of interest in the myocardium such as CERU, FBLN2, A2MG, PAPP1, CCN2, TRFL, and MFAP5 (Figure 5C). Enrichment analysis of differentially regulated proteins following LDN treatment in the SWATH dataset revealed significant enrichment of cardiovascular terms, including response to wounding, cell adhesion, and angiogenesis, suggesting that inhibition of ALK1 signaling in BMP9-treated hHiPCs results in changes in key endothelial repair pathways (Figure 5D).

Recent studies suggest that ALK1 may signal under the influence of different BMPs, e.g., BMP4/10 [3,59], and we sought to better establish that BMP9-dependent effects are due to the direct action on hHiPC ALK1. To approach this issue, we used the recombinant ligand trap protein soluble Fc-ALK1, and these data indicated that the effect of exogenously added BMP9 is blunted by Fc-ALK1, thus corroborating our previous results and confirming that BMP9-specific signaling regulates hHiPC expression of CD105, IGFBP3, ISLR and SOST via direct binding to ALK1 (Figure 5E). These data demonstrate that ALK1 signaling is necessary for BMP9 induction of transcription and secretion of novel targets IGFBP3, ISLR, and SOST.

We next examined ALK1-specific signaling via BMP9 stimulation using lentivirus-mediated knockdown of *ALK1* in hHiPC. Following transduction with lentiviral sh*ALK1* particles, we observed a significant reduction of *ALK1* mRNA expression by approximately 70% (fold-change = 0.31, p<0.01) as measured by RT-qPCR (Figure 5F). Following lentiviral knockdown and puromycin selection, we treated hHiPC with BMP9 in low serum for 24-hours. sh*ALK1* knockdown significantly reduced the BMP9 response for target proteins *CD105* (positive control), *IGFBP3*, *SOST*, *and ISLR* (Figure 5G). Secreted IGFBP3 is of particular interest due to its requirement for angiogenesis, neonatal regeneration, and cardiac development [56]. Therefore, we corroborated IGFBP3 protein secretion following *ALK1* knockdown by LC-MS/MS analysis. As expected, these experiments confirmed that BMP9 was present in the BMP9-treated CM groups and levels were consistent between treated conditions, with no detectable expression in control groups, and confirming that equivalent amount of BMP9 were added in the treated groups (Figure 5H). Importantly, endogenous BMP9 was not detected above background (Figure 5H, shNT+Vehicle and sh*ALK1*+Vehicle). Consistent with other putative ALK1-regulated proteins, we found a significant reduction in the BMP9 response of secreted IGFBP3 following *ALK1* knockdown, further confirming the role of BMP9/ALK1 hHiPC signaling in IGFBP3 expression and secretion (Figure 5H).

### Sclerostin is a candidate pro-angiogenic secretory factor of the BMP9-treated hHiPC secretome

**Figure 6.**
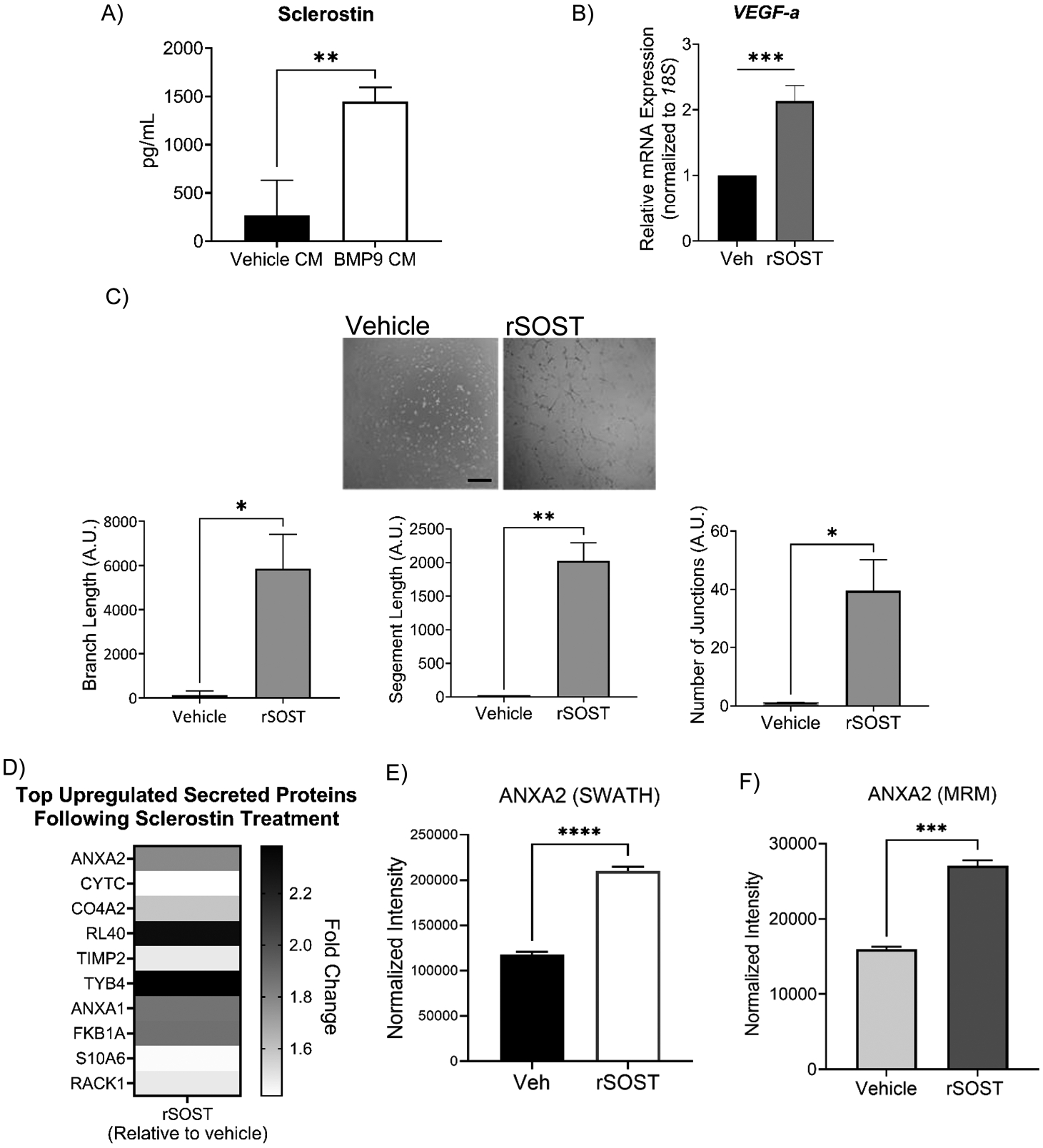
Recombinant sclerostin enhances the angiogenic response of hHiPCs, increases *VEGF-a*, and endothelial receptor ANXA2 expression *in vitro*. **A)** SOST ELISA was performed on BMP9 CM collected from hHiPC to confirm induction of SOST following BMP9 priming. Data are shown as mean ± SD and analyzed in technical triplicate using student’s t-test (n=3). **B)** *VEGF-a* expression was analyzed by RT-qPCR (n=3 independent patient-derived clones). mRNA measurements were taken in triplicate and analyzed by student’s t-test. Data were normalized to *18S*. Data are expressed as relative fold-change ± SD (** indicates p<0.01). **C)** hHiPCs (n=3) were plated on Matrigel (Corning) and incubated with SOST (100 ng/mL) or vehicle control and tube formation was measured at 6-hours. Data are expressed as mean ± SD; P-value by Student’s t-test. Scale bar, 100 μm. **D)** Unbiased LC-MS/MS SWATH analysis of rSOST-treated hHiPC secretome. Heat map of top differentially upregulated secreted proteins (FC>1.6, p<0.05). **E)** LC-MS/MS SWATH and **F)** MRM analysis of ANXA2 protein level following rSOST treatment in hHiPC. Data were normalized using MLR normalization (SWATH) or TAS normalization (MRM) and presented as mean ± SD (n=3). **** indicates p<0.0001, *** p<0.001.

We next investigated the use of EC tube formation to prioritize BMP9-target secretory proteins that may enhance hHiPC capacity for cardiac repair. Because SOST was one of the highest fold-change induced secretory proteins following BMP9 treatment and can stimulate angiogenic responses in human umbilical vein endothelial cells (HUVECs) [57], we investigated whether human recombinant SOST (rSOST) modulates hHiPC tube formation. For this experiment, and to buttress previous hHiPC expression data, we first used a SOST sandwich ELISA to confirm increased expression of SOST in the BMP9-treated CM of hHiPC (Figure 6A). We then treated hHiPC with rSOST (100 ng/mL) for 24-hours as described previously [57]. rSOST treatment resulted in increased transcription of *VEGF-a*, a master regulator of the hypoxic angiogenic response that targets cardiomyocytes, enhances EC survival, and increases capillary density at the site of myocardial infarction (Figure 6B) [58–60]. Treatment of hHiPC with rSOST in the Matrigel-based tube formation assay revealed that, at the level tested, rSOST alone induced tube formation as compared to vehicle treated controls (Figure 6C), suggesting the potential role of SOST in cardiac angiogenesis. To gain insight into regulatory effects of SOST on hHiPC, we collected the hHiPC secretome following rSOST treatment. SWATH analysis of secreted proteins revealed increased concentration of secreted proteins involved in cardiac maintenance and regeneration including annexin A2 (ANXA2, Figure 6D). SWATH data for ANXA2, shown in Figure 6E, was confirmed independently by multiple reaction monitoring LC-MS/MS (MRM, Figure 6F). We did not detect VEGF-a protein in the secretome of rSOST-treated hHiPCs as measured by SWATH and MRM mass spectrometry, suggesting that VEGF-a secreted levels, if present, may be too low for detection, and may require further follow-on studies using western blot or ELISA-based analysis. These data provide new insight into the potential significance of SOST signaling in cardiac repair via pro-angiogenic processes and regulation of endothelial proteins such as ANXA2 and others uncovered by SWATH analysis of hHiPC.

## Discussion

We have found that hHiPCs isolated from the human myocardium express ALK1 and display ALK1-dependent expression of pro-angiogenic properties in response to BMP9 treatment. Proteomic analysis of this pathway implicates specific novel secreted BMP9 and BMP10 targets that are potentially important for cardiac progenitor cell function. The identified BMP9-specific hHiPC proteins include SOST, IGFBP3, and ISLR, which our data suggest are directly regulated by ALK1 receptor expression in hHiPCs using lentiviral and small molecule inhibition of ALK1. Understanding the primary regulators of secreted factor-mediated repair has potential for therapeutic translation in the treatment of heart disease.

hHiPCs isolated directly from the epicardium of patients undergoing CABG surgery have been shown to differentiate towards endothelial cells *in vitro*, and demonstrate a highly proliferative expandability phenotype [17]. The ability of hHiPCs to form colonies is a common characteristic of therapeutic cells and identifies a subpopulation of epicardial cells that can be easily isolated from the adult heart [61–63]. In this study, we further characterize hHiPCs as a mesenchymal cell population with progenitor characteristics similar to therapeutic cardiospheres (CD105^+^, CXCL6^+^), with novel identification of expression of the endothelial receptor, ALK1. ALK1 is a BMP9/10 ligand-binding type-I receptor serine/threonine protein kinase found in endothelial and progenitor cells [3,64,65]. Many mesenchymal stem cell populations have been characterized by the presence of CD105 [66], including hHiPC [17,44]. However, the presence of easily detectable levels of ALK1 along with CD105/endoglin in hHiPC suggests that these proteins exist as a type I/Type III co-receptor complex [67] in hHiPCs. Thus, observation of variable ALK1 and CD105/endoglin levels may impact hHiPC BMP9-dependent (e.g., ALK1/Endoglin) receptor complex composition in individual patient-derived hHiPC isolates and may have implications for the efficiency of transduction of BMP9 signaling that require further investigation.

BMP9 signaling via the ALK1 receptor is a critical regulator of vascular development and angiogenesis [3,22]. BMP9/ALK1 signaling has been shown to have both anti- and pro-angiogenic effects depending on ligand concentration, biological system, and cell type [4,5,68,69]. Circulating BMP9 is negatively associated with diabetes, hypertension, and coronary artery disease (CAD) [10,11,70]. BMP9 is also increased in the ventricles and circulation of patients with heart failure, and treatment with recombinant murine BMP9 in mice improves LV function and capillary density in heart failure [6]. Other studies have demonstrated that BMP9-stimulated ALK1 signaling is critical for endothelial progenitor cell differentiation and neovascularization following ischemic injury in the hindlimb of mice [65]. Relevant to our findings, we found that in CABG patients circulating BMP9 levels are negatively correlated with number of hHiPCs and positively correlated with number of ECs isolated from the ventricular samples, suggesting that BMP9 may contribute to EC number and hHiPC fate in adult cardiac tissue. Further work is need to more fully understand the functions of BMP9 in the heart, on cardiac-derived progenitor cells, and its potential to regulate cardiac plasticity and repair in the adult myocardium.

Pro-angiogenic secreted factors are a major component of cellular therapy and the ability to promote repair [14,15,45,71]. Angiogenesis is a critical component of improved myocardial repair and can occur through direct differentiation of endogenous ECs or paracrine/autocrine signaling from exogenous stem/progenitor cell transplant [14,16,72–75]. Due to low survivability of therapeutic cells when injected into the ischemic myocardium, secreted protein-related mechanisms require more in-depth characterization and understanding [14,74]. To date, no study has investigated the importance of the ALK1 receptor and the role of this pathway in therapeutic human cell lines. Our studies are the first to reveal that pre-treating hHiPCs with BMP9 improves tube formation *in vitro* through paracrine and autocrine response mechanisms. This BMP9/10 concentration was chosen due to relevance of circulating BMP9/10 in the blood (ranging from 2–12 ng/mL) and previous EC studies [3,22,23,76]. These regulatory mechanisms include ALK1-specific upregulation of SOST, IGFBP3, and ISLR by BMP9. IGFBP3 is also upregulated upon BMP10 stimulation suggesting that regulation of SOST and ISLR is ligand dependent, further emphasizing our study of BMP9/SOST axis in angiogenesis. Depleting ALK1 by lentiviral knockdown or inhibiting ALK1 protein signaling through Fc-ALK1 ligand trap or small molecule inhibitors all result in significant downregulation of SOST, IGFBP3, and ISLR following BMP9 treatment on the transcriptional and secreted protein level. Small molecule inhibition in hHiPCs and collected conditioned media revealed enrichment for pathways involved in endothelial cell function such as cell adhesion and angiogenesis. These data indicate preferential changes to the hHiPC secretome that may be involved in key reparative pathways involved in EC maintenance. Additional secreted proteins that decreased in concentration upon ALK1 inhibition, such as CERU, FBLN2, A2MG, PAPP1, CCN2, TRFL, and MFAP5 suggest new roles for these proteins as novel mediators of ALK1 function in cardiac progenitor cells and will require further investigation for their potential roles in angiogenesis mediated by ALK1.

The present study focused on the most differentially expressed proteins; SOST, IGFBP3, and ISLR, induced by BMP9 and characterized the ALK1 signaling pathway in patient-derived hHiPC. These data highlight the importance of BMP9/ALK1 via regulation of SOST, IGFBP3, and ISLR in the potential beneficial effects of the cardiac secretome *in vitro*.

Understanding how secreted hHiPC proteins regulate cardiac repair may lead to the development of tailored cell-based therapies as well as cell-free treatments. Our studies highlight that there may be many secretome factors involved in different repair processes, and we chose SOST as the first BMP9 target for investigation due to previous studies of SOST in HUVEC angiogenesis, its’ large fold-change induction (FC>10), access to commercially available recombinant human SOST, and its role as a known WNT antagonist. SOST is a WNT inhibitor that has been shown to have important regulatory roles in bone formation, osteoblast differentiation, vasculature calcification and angiogenesis [24,77,78]. WNT signaling is active during cardiac development and quiescent in adult life where it can be reactivated during myocardial injury [79]. This property makes inhibiting WNT signaling during myocardial infarction a potential therapeutic target [80]. An antibody to SOST, romosozumab, is used to treat osteoporosis in patients, but may have negative effects on cardiovascular function [78,81]. Several studies have demonstrated the protective effect of SOST on vascular calcification and endothelial function [77,82]. Specifically in the heart, murine studies indicate that sclerostin may oppose cardiac tissue and vessel calcification [77]. Similar *in vitro* studies in human cells have confirmed this protective effect [83]. SOST functions by binding to LRP5/6 and inhibiting canonical WNT/β-catenin signaling, a prominent targeted pathway of interest in cardiac fibrosis, regeneration, and angiogenesis [84]. Although SOST has been shown to have pro-angiogenic effects *in vitro*, recent evidence suggests that SOST is increased in the heart following MI and global overexpression *SOST* 24-hours post-MI *in vivo* aggravates cardiac remodeling and angiogenesis in mice [85]. These observations suggest that dose, timing, and affected cell type may contribute to contradictory findings involving SOST in the heart.

In our studies, we found that SOST is upregulated by BMP9/ALK1 in hHiPCs, and that rSOST (100 ng/mL) treatment improves tube formation of hHiPCs *in vitro*. This concentration of sclerostin is higher than reported circulating levels (~1 ng/mL), but discrepancies have been reported across different commercial assays, and increases drastically with age [86,87]. We also found that treatment of hHiPC with human recombinant SOST enhances regulation of *VEGF-a* along with other novel angiogenic/cardiac maintenance proteins such as annexin A2 (ANXA2). ANXA2 is part of the calcium-dependent phospholipid-binding protein family and is highly expressed in ECs to regulate their function. ANXA2 expression regulates several angiogenic EC processes such as migration, proliferation, and capillary sprouting [88,89]. Importantly, ANXA2 has a prominent role in stem cell fate and function regulating regenerative processes such as stem cell homing, adhesion, engraftment, and differentiation [90]. ANXA2 also interacts with CXCL12 (SDF1), a BMP9-inducible protein in endothelial cells [55] and a well-defined homing molecule identified for improved stem cell treatments in heart disease [74,91–93]. Our studies are the first to find that ANXA2 is regulated by SOST. More study is needed to determine the direct role of a potential ALK1/SOST autocrine axis in different cell types and the potential of SOST as a pro-angiogenic treatment in MI. Treating hHiPCs with BMP9 may also be critical for regulation of SOST expression and for further development of hHiPCs as a therapeutic treatment in ischemic heart disease.

Additional regulated targets of BMP9/ALK1 signaling in hHiPC are of potential interest for further study. ISLR (meflin) is a novel mesenchymal stem cell marker that is involved in cardiac repair through inhibiting fibrosis and interacting with BMP7 to suppress TGFβ signaling [94,95]. ISLR is a secreted factor that has been shown to regulate WNT signaling during skeletal muscle regeneration [96]. ISLR is also downregulated in aged mouse hearts suggesting the potential role of this secreted protein in cardiac regeneration and repair [94]. Our data are the first to find that ISLR is an upregulated secreted target of BMP9/ALK1 signaling. Given the potential therapeutic role of ISLR, further studies should investigate the paracrine role of ISLR signaling in hHiPC, with and without BMP9, to determine the distinct role secreted ISLR may have in pro-angiogenic and regenerative mechanisms. Other potentially relevant secreted factors found in the BMP9 treated hHiPC conditioned medium, including IGFBP3, CXCL5, CXCL1, and IL6, have been found to be important in the paracrine properties of cardiac cells and during neonatal heart regeneration [45,97–100] and merit further study in the class of cardiac progenitor cells with angiogenic potential represented by hHiPC.

Optimizing cell therapy holds promise in the treatment of heart disease and important studies have highlighted the need for understanding cell mechanisms of secreted protein-dependent responses following cell treatment. Further investigation of the BMP9-treated hHiPC secretome will be needed to elucidate which proteins are the primary regulators of paracrine induced healing, such as SOST, IGFBP3, or ISLR *in vivo*. For example, the potential SOST target, ANXA2, plays a role in protective vascularization following myocardial infarction, interacting with macrophage YAP and integrin signaling [101] as well as PI3K/AKT signaling [102]. Further, SOST target, thymosin beta-4 (TYB4), impedes stem cell differentiation, elevating self-renewal markers such as *OCT4* and *NANOG* [103]. Such studies of secretome targets can be important clinically as patients treated with or without cells may need a specific cocktail of proteins to maximize the benefit of cardiac repair and further improve symptoms and heart function over-time. Furthermore, understanding the potential interplay between IGFBP3, ISLR, SOST, and paracrine/autocrine response is a pivotal step in the identification of new or improved drug targets for the treatment of heart disease. Determining whether these target proteins are necessary for angiogenesis or EC function in the BMP9/ALK1 signaling response will be critical next steps of study and our studies provide the basis for these investigations in hHiPCs and other ALK1+ cell-types.

## Conclusion

In summary, the data provided by this study suggest the importance of BMP9/ALK1 signaling in hHiPC mediated secretome response and angiogenesis with identification of novel regulatory targets, and potential significance of BMP9/ALK1 in successful cell-therapeutic treatment in MI. As such, the present work identifies potential functional protein expression signatures of hHiPC that will be useful in the design and interpretation of *in vivo* experimental myocardial infarction experiments.

## Figures and Tables

**Table 1.** Subject demographics.

Characteristic	N=24
Age years, mean (SD)	62 (12)
Sex, n (%) female	6 (25%)
BMI, mean (SD)	31 (6)
Diabetic, n (%)	12 (50%)
Preoperative HbA1C, mean (SD)	7 (2)
Heart Failure, n (%)	2 (8%)
Smoker, n (%)	18 (75%)

**Table 2.** List of primers used for RT-qPCR.

Species	Gene	Forward (5′ -> 3′)	Reverse (5′ -> 3′)
Hu	*ALK1*	ACTCACAGGGCAGCGATTAC	CATTGGGCACCACATCATAG
Hu	*CD105*	AGCCCCACAAGTCTTGCAG	GCTAGTGGTATATGTCACCTCGC
Hu	*18S*	ATCCCTGAAAAGTTCCAGCA	CCCTCTTGGTGAGGTCAATG
Hu	*SOST*	CAGGCGTTCAAGAATGATGC	CTTTGGTCTCAAAGGGGTGG
Hu	*ISLR*	CGACTGTGGGGAAAAGTATGG	GGCTCAGTGTAGTCACATTGG
Hu	*CXCL5*	AGCTGCGTTGCGTTTGTTTAC	TGGCGAACACTTGCAGATTAC
Hu	*VEGF-a*	AGGGCAGAATCATCACGAAGT	AGGGTCTCGATTGGATGGCA
Hu	*NANOG*	GAGGTGGCAGAAAAACAACT	CTGGGGTAGGTAGGTGCTGA
Hu	*OCT4*	GAGAACCGAGTGAGAGGCAAC	CTTCTGGCGCCGGTTACAGA
Hu	*SOX2*	GCAACGGCAGCTACAGCATGAT	CTGCGAGTAGGACATGCTGTA
Hu	*BMI1*	GTCATGTATGAGGAGGAACCTT	CGAACTCTGTATTTCAATGGAAGT

**Table 3.** List of top upregulated BMP9-treated versus control proteins (sorted by p-value).

Peak Name	p-value	Fold Change
IGF2	9.53E-05	1.8
FBLN5	0.00064	1.9
**IBP3**	**0.00088**	**2.0**
CATD	0.0031	1.7
FETA	0.00412	1.6
CCN2	0.00415	2.8
PPIA	0.00423	1.7
POSTN	0.00584	2.0
FBLN2	0.00724	2.8
MYZAP	0.0077	1.5
AMBP	0.01013	2.0
PTGDS	0.0107	2.2
SODM	0.01136	2.5
**ISLR**	**0.01142**	**3.1**
COTL1	0.01312	1.9
CD14	0.0133	2.5
TSP3	0.01538	2.0
SYNC	0.01694	8.7
ITIH3	0.01887	1.5
TSP1	0.02017	2.1
HNRPQ	0.02129	2.1
**SOST**	**0.0235**	**2.7**
BGH3	0.02361	1.8
NID2	0.02455	1.9
MGP	0.02743	2.8

## Abbreviations

ALK1: Activin A Receptor-like Kinase 1
BMP9: Bone Morphogenetic Protein-9
CD105: Endoglin
CM: Conditioned Media
CPC: Cardiac Progenitor Cell
DTT: Dithiothreitol
EGM-2: Endothelial Cell Growth Medium-2
FBS: Fetal Bovine Serum
hHiPC: Human Highly Proliferative Cells
HREC: Human Retinal Endothelial Cells
IAA: Iodoacetamide
IGFBP3: Insulin-like Growth Factor Binding Protein-3
LC-MS/MS: Liquid Chromatography with Tandem Mass Spectrometry
M-199: Medium 199
NT: Non-Targeting Scrambled
RT-qPCR: Real-time Quantitative PCR
SOST: Sclerostin
SWATH: Sequential Windowing of All Theoretical Mass Spectra
CABG: Coronary Artery Bypass Graft
